# Supplementary figures and images for: Functional Analysis of the Expanded Phosphodiesterase Gene Family in Toxoplasma gondii Tachyzoites
Source: mSphere. 2022 Feb 2;7(1):e00793-21. doi: 10.1128/msphere.00793-21 (PMC8809380; doi:10.1128/msphere.00793-21)

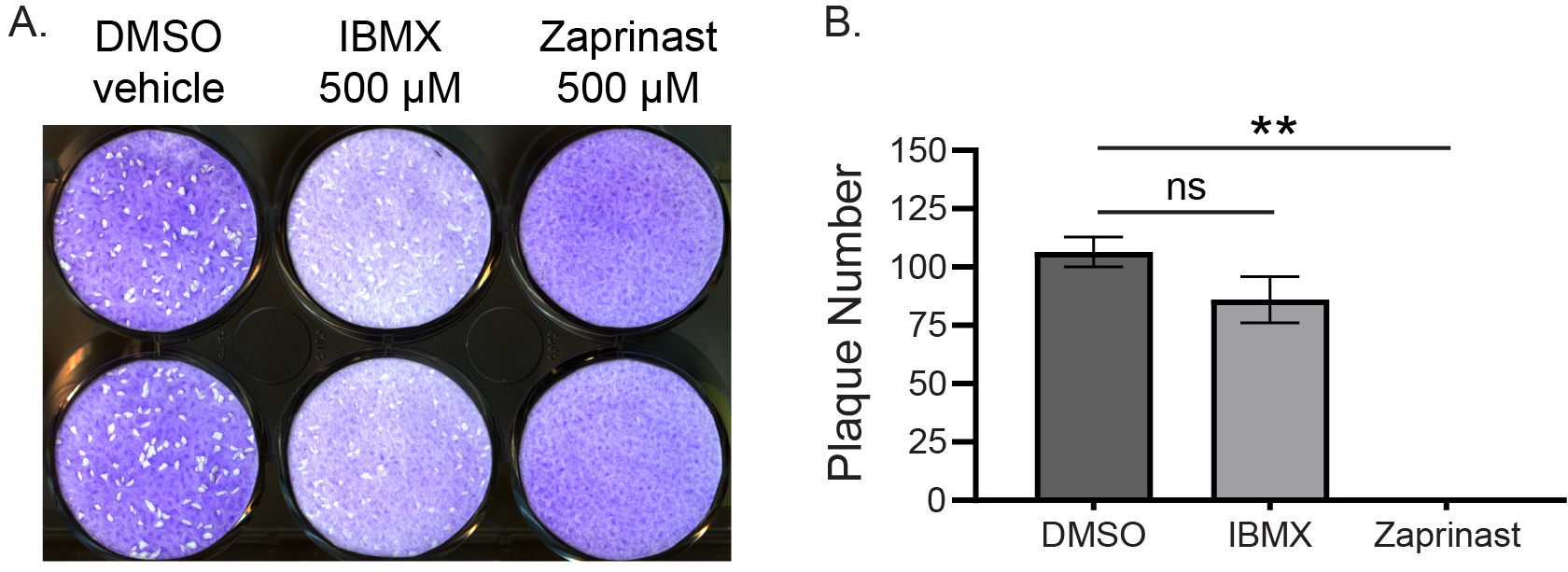

Supplement: FIG S1 [file msphere.00793-21-sf001.tif]

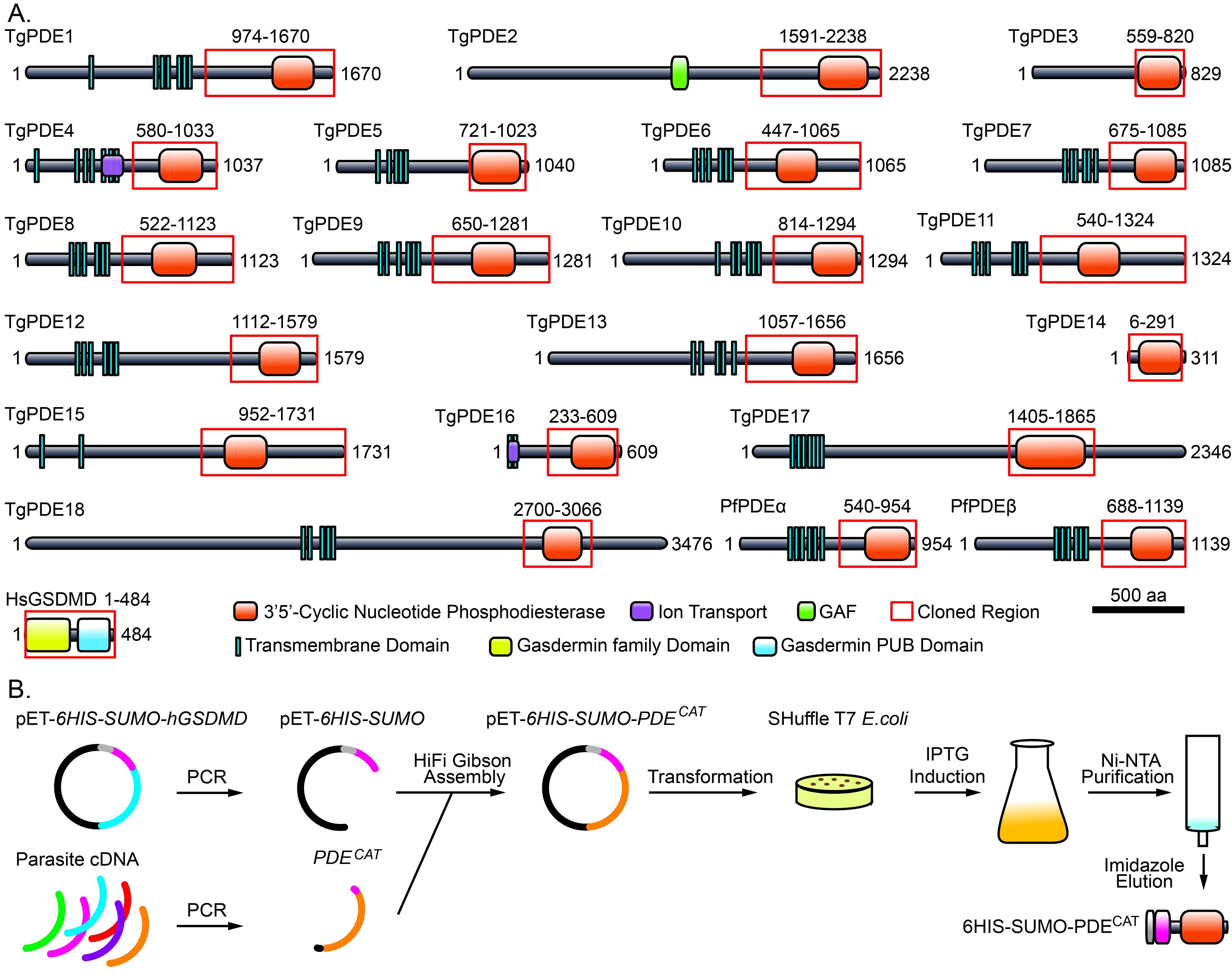

Supplement: FIG S2 [file msphere.00793-21-sf002.tif]

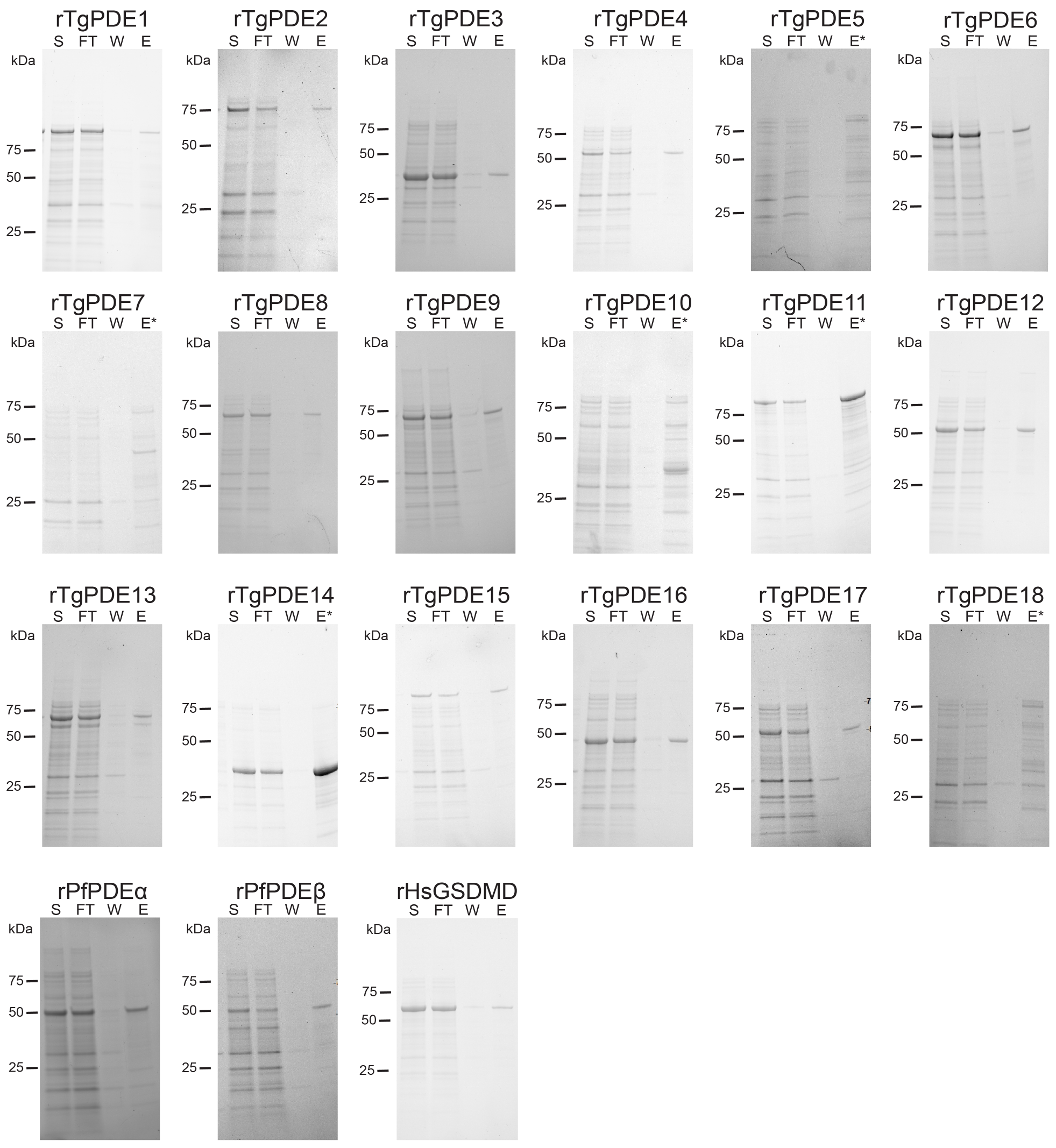

Supplement: FIG S3 [file msphere.00793-21-sf003.tif]

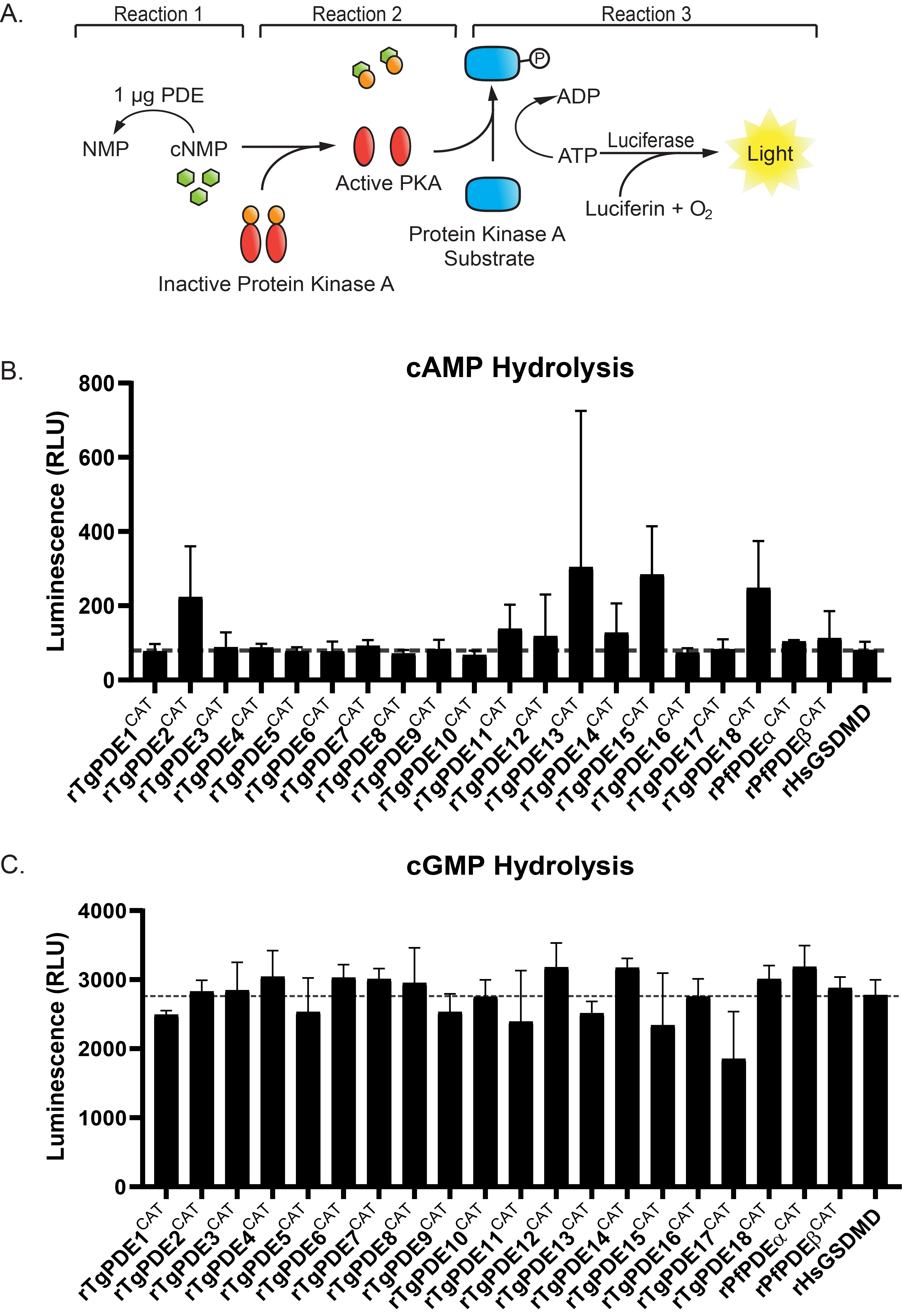

Supplement: FIG S4 [file msphere.00793-21-sf004.tif]

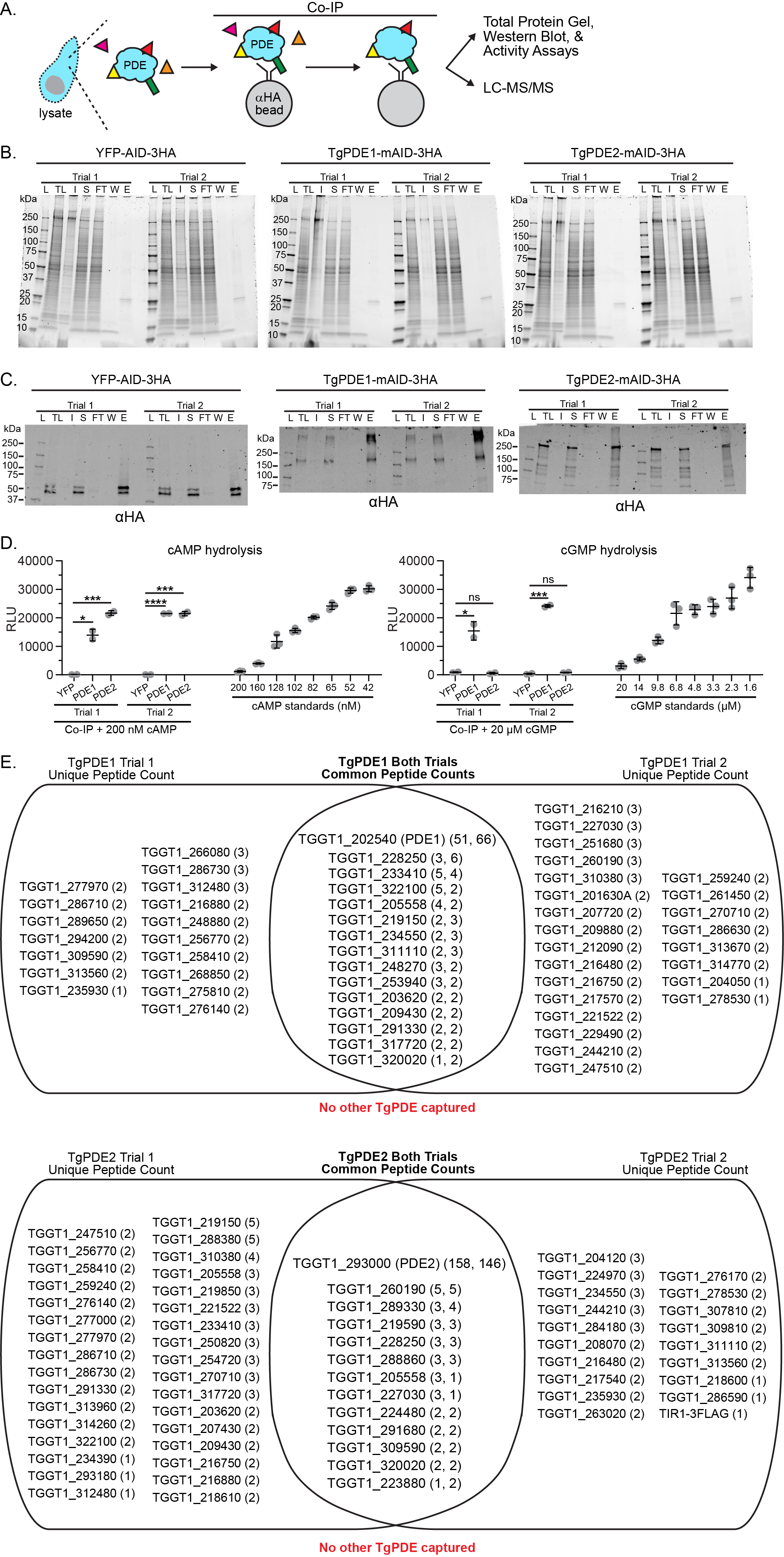

Supplement: FIG S5 [file msphere.00793-21-sf005.tif]
