# Supplementary material for: Functional Analysis of the Expanded Phosphodiesterase Gene Family in Toxoplasma gondii Tachyzoites
Source: mSphere. 2022 Feb 2;7(1):e00793-21. doi: 10.1128/msphere.00793-21 (PMC8809380; doi:10.1128/msphere.00793-21)
Supplement: TABLE S1 [file msphere.00793-21-st001.docx]

**Table S1** *Toxoplasma* strains used in this study.

| **Strain Name** | **Genotype** | **Source** |
| --- | --- | --- |
| RH | RH | ATCC #50838 |
| GT1 | GT1 | L. David Sibley Laboratory (WUSTL) |
| RH TIR1-3FLAG | RH*ΔhxgprtΔku80; TUB1:TIR1-3FLAG, SAG1:CAT* | Brown et al., 2017; Long et al., 2017 |
| RH PDE1-mAID-3HA | RH*ΔhxgprtΔku80; TUB1:TIR1-3FLAG, SAG1:CAT; PDE1-mAID-3HA, DHFR-TS:HXGPRT* | This work |
| RH PDE2-mAID-3HA | RH*ΔhxgprtΔku80; TUB1:TIR1-3FLAG, SAG1:CAT; PDE2-mAID-3HA, DHFR-TS:HXGPRT* | This work |
| RH PDE3-mAID-3HA | RH*ΔhxgprtΔku80; TUB1:TIR1-3FLAG, SAG1:CAT; PDE3-mAID-3HA, DHFR-TS:HXGPRT* | This work |
| RH PDE4-mAID-3HA | RH*ΔhxgprtΔku80; TUB1:TIR1-3FLAG, SAG1:CAT; PDE4-mAID-3HA, DHFR-TS:HXGPRT* | This work |
| RH PDE5-mAID-3HA | RH*ΔhxgprtΔku80; TUB1:TIR1-3FLAG, SAG1:CAT; PDE5-mAID-3HA, DHFR-TS:HXGPRT* | This work |
| RH PDE6-mAID-3HA | RH*ΔhxgprtΔku80; TUB1:TIR1-3FLAG, SAG1:CAT; PDE6-mAID-3HA, DHFR-TS:HXGPRT* | This work |
| RH PDE7-mAID-3HA | RH*ΔhxgprtΔku80; TUB1:TIR1-3FLAG, SAG1:CAT; PDE7-mAID-3HA, DHFR-TS:HXGPRT* | This work |
| RH PDE8-mAID-3HA | RH*ΔhxgprtΔku80; TUB1:TIR1-3FLAG, SAG1:CAT; PDE8-mAID-3HA, DHFR-TS:HXGPRT* | This work |
| RH PDE9-mAID-3HA | RH*ΔhxgprtΔku80; TUB1:TIR1-3FLAG, SAG1:CAT; PDE9-mAID-3HA, DHFR-TS:HXGPRT* | This work |
| RH PDE10-mAID-3HA | RH*ΔhxgprtΔku80; TUB1:TIR1-3FLAG, SAG1:CAT; PDE10-mAID-3HA, DHFR-TS:HXGPRT* | This work |
| RH PDE11-mAID-3HA | RH*ΔhxgprtΔku80; TUB1:TIR1-3FLAG, SAG1:CAT; PDE11-mAID-3HA, DHFR-TS:HXGPRT* | This work |
| RH PDE12-mAID-3HA | RH*ΔhxgprtΔku80; TUB1:TIR1-3FLAG, SAG1:CAT; PDE12-mAID-3HA, DHFR-TS:HXGPRT* | This work |
| RH PDE13-mAID-3HA | RH*ΔhxgprtΔku80; TUB1:TIR1-3FLAG, SAG1:CAT; PDE13-mAID-3HA, DHFR-TS:HXGPRT* | This work |
| RH PDE14-mAID-3HA | RH*ΔhxgprtΔku80; TUB1:TIR1-3FLAG, SAG1:CAT; PDE14-mAID-3HA, DHFR-TS:HXGPRT* | This work |
| RH PDE15-mAID-3HA | RH*ΔhxgprtΔku80; TUB1:TIR1-3FLAG, SAG1:CAT; PDE15-mAID-3HA, DHFR-TS:HXGPRT* | This work |
| RH PDE16-mAID-3HA | RH*ΔhxgprtΔku80; TUB1:TIR1-3FLAG, SAG1:CAT; PDE16-mAID-3HA, DHFR-TS:HXGPRT* | This work |
| RH PDE17-mAID-3HA | RH*ΔhxgprtΔku80; TUB1:TIR1-3FLAG, SAG1:CAT; PDE17-mAID-3HA, DHFR-TS:HXGPRT* | This work |
| RH PDE18-mAID-3HA | RH*ΔhxgprtΔku80; TUB1:TIR1-3FLAG, SAG1:CAT; PDE18-mAID-3HA, DHFR-TS:HXGPRT* | This work |
| RH YFP-AID-3HA | RH*ΔhxgprtΔku80; TUB1:TIR1-3FLAG, SAG1:CAT; TUB1:YFP-AID-3HA, DHFR-TS:HXGPRT* | Long et al., 2017 |
