# Supplementary material for: Functional Analysis of the Expanded Phosphodiesterase Gene Family in Toxoplasma gondii Tachyzoites
Source: mSphere. 2022 Feb 2;7(1):e00793-21. doi: 10.1128/msphere.00793-21 (PMC8809380; doi:10.1128/msphere.00793-21)
Supplement: TABLE S2 [file msphere.00793-21-st002.docx]

**Table S2** Plasmids used in this study.

| **p#** | **Plasmid Name** | **Description** | **Usage** | **Source** |
| --- | --- | --- | --- | --- |
| p1 | p*TUB1:YFP-mAID-3HA, DHFR-TS:HXGPRT* | *YFP-mAID-3HA* fusion driven by a minimal *TgTUB1* promoter with an *TgHXGPRT* drug selectable marker. | PCR template for generating *TgPDE-mAID-3HA* tagging amplicons. | Brown et al. 2017 |
| p2 | p*SAG1:Cas9-GFP, U6:_gRNA* | Linear *SpCas9-GFP*, *gRNA* (no protospacer) destination vector | Linear *Cas9-GFP, gRNA* (no protospacer) destination vector for inserting *GOI protospacer* by ssDNA (59-60mer) HiFi assembly | Shen et al. 2014 |
| p3 | p*SAG1:Cas9-GFP, U6:sg202540(PDE1) 3’UTR* | *Streptococcus pyogenes* *Cas9* fused to *GFP* driven by a *TgSAG1* promoter and *CRISPR sgRNA targeting TgPDE1 3’ UTR* driven from a *Pol III TgU6* promoter | Co-transfection with *TgPDE1-mAID-3HA, DHFR:HXGPRT* amplicon for CRISPR/Cas9 tagging C-terminus of TgPDE1. | This work |
| p4 | p*SAG1:Cas9-GFP, U6:sg293000(PDE2) 3’UTR* | *Streptococcus pyogenes* *Cas9* fused to *GFP* driven by a *TgSAG1* promoter and *CRISPR sgRNA targeting TgPDE2 3’ UTR* driven from a *Pol III TgU6* promoter | Co-transfection with *TgPDE2-mAID-3HA, DHFR:HXGPRT* amplicon for CRISPR/Cas9 tagging C-terminus of TgPDE2. | This work |
| p5 | p*SAG1:Cas9-GFP, U6:sg233065(PDE3) 3’UTR* | *Streptococcus pyogenes* *Cas9* fused to *GFP* driven by a *TgSAG1* promoter and *CRISPR sgRNA targeting TgPDE3 3’ UTR* driven from a *Pol III TgU6* promoter | Co-transfection with *TgPDE3-mAID-3HA, DHFR:HXGPRT* amplicon for CRISPR/Cas9 tagging C-terminus of TgPDE3. | This work |
| p6 | p*SAG1:Cas9-GFP, U6:sg229405(PDE4) 3’UTR* | *Streptococcus pyogenes* *Cas9* fused to *GFP* driven by a *TgSAG1* promoter and *CRISPR sgRNA targeting TgPDE4 3’ UTR* driven from a *Pol III TgU6* promoter | Co-transfection with *TgPDE4-mAID-3HA, DHFR:HXGPRT* amplicon for CRISPR/Cas9 tagging C-terminus of TgPDE4. | This work |
| p7 | p*SAG1:Cas9-GFP, U6:sg220420(PDE5) 3’UTR* | *Streptococcus pyogenes* *Cas9* fused to *GFP* driven by a *TgSAG1* promoter and *CRISPR sgRNA targeting TgPDE5 3’ UTR* driven from a *Pol III TgU6* promoter | Co-transfection with *TgPDE5-mAID-3HA, DHFR:HXGPRT* amplicon for CRISPR/Cas9 tagging C-terminus of *Tg*PDE5. | This work |
| p8 | p*SAG1:Cas9-GFP, U6:sg266920(PDE6) 3’UTR* | *Streptococcus pyogenes* *Cas9* fused to *GFP* driven by a *TgSAG1* promoter and *CRISPR sgRNA targeting TgPDE6 3’ UTR* driven from a *Pol III TgU6* promoter | Co-transfection with *TgPDE6-mAID-3HA, DHFR:HXGPRT* amplicon for CRISPR/Cas9 tagging C-terminus of TgPDE6. | This work |
| p9 | p*SAG1:Cas9-GFP, U6:sg280410(PDE7) 3’UTR* | *Streptococcus pyogenes* *Cas9* fused to *GFP* driven by a *TgSAG1* promoter and *CRISPR sgRNA targeting TgPDE7 3’ UTR* driven from a *Pol III TgU6* promoter | Co-transfection with *TgPDE7-mAID-3HA, DHFR:HXGPRT* amplicon for CRISPR/Cas9 tagging C-terminus of TgPDE7. | This work |
| p10 | p*SAG1:Cas9-GFP, U6:sg318675(PDE8) 3’UTR* | *Streptococcus pyogenes* *Cas9* fused to *GFP* driven by a *TgSAG1* promoter and *CRISPR sgRNA targeting TgPDE8 3’ UTR* driven from a *Pol III TgU6* promoter | Co-transfection with *TgPDE8-mAID-3HA, DHFR:HXGPRT* amplicon for CRISPR/Cas9 tagging C-terminus of TgPDE8. | This work |
| p11 | p*SAG1:Cas9-GFP, U6:sg241880(PDE9) 3’UTR* | *Streptococcus pyogenes* *Cas9* fused to *GFP* driven by a *TgSAG1* promoter and *CRISPR sgRNA targeting TgPDE9 3’ UTR* driven from a *Pol III TgU6* promoter | Co-transfection with *TgPDE9-mAID-3HA, DHFR:HXGPRT* amplicon for CRISPR/Cas9 tagging C-terminus of TgPDE9. | This work |
| p12 | p*SAG1:Cas9-GFP, U6:sg272650(PDE10) 3’UTR* | *Streptococcus pyogenes* *Cas9* fused to *GFP* driven by a *TgSAG1* promoter and *CRISPR sgRNA targeting TgPDE10 3’ UTR* driven from a *Pol III TgU6* promoter | Co-transfection with *TgPDE10-mAID-3HA, DHFR:HXGPRT* amplicon for CRISPR/Cas9 tagging C-terminus of TgPDE10. | This work |
| p13 | p*SAG1:Cas9-GFP, U6:sg224840(PDE11) 3’UTR* | *Streptococcus pyogenes* *Cas9* fused to *GFP* driven by a *TgSAG1* promoter and *CRISPR sgRNA targeting TgPDE11 3’ UTR* driven from a *Pol III TgU6* promoter | Co-transfection with *TgPDE11-mAID-3HA, DHFR:HXGPRT* amplicon for CRISPR/Cas9 tagging C-terminus of TgPDE11. | This work |
| p14 | p*SAG1:Cas9-GFP, U6:sg310520(PDE12) 3’UTR* | *Streptococcus pyogenes* *Cas9* fused to *GFP* driven by a *TgSAG1* promoter and *CRISPR sgRNA targeting TgPDE12 3’ UTR* driven from a *Pol III TgU6* promoter | Co-transfection with *TgPDE12-mAID-3HA, DHFR:HXGPRT* amplicon for CRISPR/Cas9 tagging C-terminus of TgPDE12. | This work |
| p15 | p*SAG1:Cas9-GFP, U6:sg257080(PDE13) 3’UTR* | *Streptococcus pyogenes* *Cas9* fused to *GFP* driven by a *TgSAG1* promoter and *CRISPR sgRNA targeting TgPDE13 3’ UTR* driven from a *Pol III TgU6* promoter | Co-transfection with *TgPDE13-mAID-3HA, DHFR:HXGPRT* amplicon for CRISPR/Cas9 tagging C-terminus of TgPDE13. | This work |
| p16 | p*SAG1:Cas9-GFP, U6:sg228500(PDE14) 3’UTR* | *Streptococcus pyogenes* *Cas9* fused to *GFP* driven by a *TgSAG1* promoter and *CRISPR sgRNA targeting TgPDE14 3’ UTR* driven from a *Pol III TgU6* promoter | Co-transfection with *TgPDE14-mAID-3HA, DHFR:HXGPRT* amplicon for CRISPR/Cas9 tagging C-terminus of TgPDE14. | This work |
| p17 | p*SAG1:Cas9-GFP, U6:sg233040(PDE15) 3’UTR* | *Streptococcus pyogenes* *Cas9* fused to *GFP* driven by a *TgSAG1* promoter and *CRISPR sgRNA targeting TgPDE15 3’ UTR* driven from a *Pol III TgU6* promoter | Co-transfection with *TgPDE15-mAID-3HA, DHFR:HXGPRT* amplicon for CRISPR/Cas9 tagging C-terminus of TgPDE15. | This work |
| p18 | p*SAG1:Cas9-GFP, U6:sg258508(PDE16) 3’UTR* | *Streptococcus pyogenes* *Cas9* fused to *GFP* driven by a *TgSAG1* promoter and *CRISPR sgRNA targeting TgPDE16 3’ UTR* driven from a *Pol III TgU6* promoter | Co-transfection with *TgPDE16-mAID-3HA, DHFR:HXGPRT* amplicon for CRISPR/Cas9 tagging C-terminus of TgPDE16. | This work |
| p19 | p*SAG1:Cas9-GFP, U6:sg257945(PDE17) 3’UTR* | *Streptococcus pyogenes* *Cas9* fused to *GFP* driven by a *TgSAG1* promoter and *CRISPR sgRNA targeting TgPDE17 3’ UTR* driven from a *Pol III TgU6* promoter | Co-transfection with *TgPDE17-mAID-3HA, DHFR:HXGPRT* amplicon for CRISPR/Cas9 tagging C-terminus of TgPDE17. | This work |
| p20 | p*SAG1:Cas9-GFP, U6:sg226755(PDE18) 3’UTR* | *Streptococcus pyogenes* *Cas9* fused to *GFP* driven by a *TgSAG1* promoter and *CRISPR sgRNA targeting TgPDE18 3’ UTR* driven from a *Pol III TgU6* promoter | Co-transfection with *TgPDE18-mAID-3HA, DHFR:HXGPRT* amplicon for CRISPR/Cas9 tagging C-terminus of TgPDE18. | This work |
| p21 | pET-*6HIS-SUMO-hGSDMD* | *E. coli* expression vector with a *KanR* drug selectable marker. Addgene #111559 | Subcloning. Negative control protein for PDE assays | Addgene; Kambara et al. 2018 |
| p22 | pET-*6HIS*-*SUMO*-*TgPDE1*^974-1670^ | pET-*6HIS*-*SUMO*-*TgPDE1*^974-1670^ driven by a *T7* promoter with a *KanR* drug selectable marker. | Expression and purification of recombinant TgPDE1^974-1670^ from *E. coli*. | This work |
| p23 | pET-*6HIS*-*SUMO*-*TgPDE2*^1591-2238^ | pET-*6HIS*-*SUMO*-*TgPDE2*^1591-2238^ driven by a *T7* promoter with a *KanR* drug selectable marker. | Expression and purification of recombinant TgPDE2^1591-2238^ from *E. coli*. | This work |
| p24 | pET-*6HIS*-*SUMO*-*TgPDE3*^559-820^ | pET-*6HIS*-*SUMO*-*TgPDE3*^559-820^ driven by a *T7* promoter with a *KanR* drug selectable marker. | Expression and purification of recombinant TgPDE3^559-820^ from *E. coli*. | This work |
| p25 | pET-*6HIS*-*SUMO*-*TgPDE4*^580-1033^ | pET-*6HIS*-*SUMO*-*TgPDE4*^580-1033^ driven by a *T7* promoter with a *KanR* drug selectable marker. | Expression and purification of recombinant TgPDE4^580-1033^ from *E. coli*. | This work |
| p26 | pET-*6HIS*-*SUMO*-*TgPDE5*^721-1023^ | pET-*6HIS*-*SUMO*-*TgPDE5*^721-1023^ driven by a *T7* promoter with a *KanR* drug selectable marker. | Expression and purification of recombinant TgPDE5^721-1023^ from *E. coli*. | This work |
| p27 | pET-*6HIS*-*SUMO*-*TgPDE6*^447-1065^ | pET-*6HIS*-*SUMO*-*TgPDE6*^447-1065^ driven by a *T7* promoter with a KanR drug selectable marker. | Expression and purification of recombinant TgPDE6^447-1065^ from *E. coli*. | This work |
| p28 | pET-*6HIS*-*SUMO*-*TgPDE7*^675-1085^ | pET-*6HIS*-*SUMO*-*TgPDE7*^675-1085^ driven by a *T7* promoter with a *KanR* drug selectable marker. | Expression and purification of recombinant TgPDE7^675-1085^ from *E. coli*. | This work |
| p29 | pET-*6HIS*-*SUMO*-*TgPDE8*^522-1123^ | pET-*6HIS*-*SUMO*-*TgPDE8*^522-1123^ driven by a *T7* promoter with a *KanR* drug selectable marker. | Expression and purification of recombinant TgPDE8^522-1123^ from *E. coli*. | This work |
| p30 | pET-*6HIS*-*SUMO*-*TgPDE9*^650-1281^ | pET-*6HIS*-*SUMO*-*TgPDE9*^650-1281^ driven by a *T7* promoter with a *KanR* drug selectable marker. | Expression and purification of recombinant TgPDE9^650-1281^ from *E. coli*. | This work |
| p31 | pET-*6HIS*-*SUMO*-*TgPDE10*^814-1294^ | pET-*6HIS*-*SUMO*-*TgPDE10*^814-1294^ driven by a *T7* promoter with a *KanR* drug selectable marker. | Expression and purification of recombinant TgPDE10^814-1294^ from *E. coli*. | This work |
| p32 | pET-*6HIS*-*SUMO*-*TgPDE11*^540-1324^ | pET-*6HIS*-*SUMO*-*TgPDE11*^540-1324^ driven by a *T7* promoter with a *KanR* drug selectable marker. | Expression and purification of recombinant TgPDE11^540-1324^ from *E. coli*. | This work |
| p33 | pET-*6HIS*-*SUMO*-*TgPDE12*^1112-1579^ | pET-*6HIS*-*SUMO*-*TgPDE12*^1112-1579^ driven by a *T7* promoter with a *KanR* drug selectable marker. | Expression and purification of recombinant TgPDE12^1112-1579^ from *E. coli*. | This work |
| p34 | pET-*6HIS*-*SUMO*-*TgPDE13*^1057-1656^ | pET-*6HIS*-*SUMO*-*TgPDE13*^1057-1656^ driven by a *T7* promoter with a *KanR* drug selectable marker. | Expression and purification of recombinant TgPDE13^1057-1656^ from *E. coli*. | This work |
| p35 | pET-*6HIS*-*SUMO*-*TgPDE14*^6-291^ | pET-*6HIS*-*SUMO*-*TgPDE14*^6-291^ driven by a *T7* promoter with a *KanR* drug selectable marker. | Expression and purification of recombinant TgPDE14^6-291^ from *E. coli*. | This work |
| p36 | pET-*6HIS*-*SUMO*-*TgPDE15*^952-1731^ | pET-*6HIS*-*SUMO*-*TgPDE15*^952-1731^ driven by a *T7* promoter with a *KanR* drug selectable marker. | Expression and purification of recombinant TgPDE15^952-1731^ from *E. coli*. | This work |
| p37 | pET-*6HIS*-*SUMO*-*TgPDE16*^233-609^ | pET-*6HIS*-*SUMO*-*TgPDE16*^233-609^ driven by a *T7* promoter with a *KanR* drug selectable marker. | Expression and purification of recombinant TgPDE16^233-609^ from *E. coli*. | This work |
| p38 | pET-*6HIS*-*SUMO*-*TgPDE17*^1405-1865^ | pET-*6HIS*-*SUMO*-*TgPDE17*^1405-1865^ driven by a *T7* promoter with a *KanR* drug selectable marker. | Expression and purification of recombinant TgPDE17^1405-1865^ from *E. coli*. | This work |
| p39 | pET-*6HIS*-*SUMO*-*TgPDE18*^2700-3066^ | pET-*6HIS*-*SUMO*-*TgPDE18*^2700-3066^ driven by a *T7* promoter with a *KanR* drug selectable marker. | Expression and purification of recombinant TgPDE18^2700-3066^ from *E. coli*. | This work |
| p40 | pET-*6HIS*-*SUMO*-*PfPDEα*^540-954^ | pET-*6HIS*-*SUMO*-*PfPDEα*^540-954^ driven by a *T7* promoter with a *KanR* drug selectable marker. | Expression and purification of recombinant PfPDEα^540-954^ from *E. coli*. | This work |
| p41 | pET-*6HIS*-*SUMO*-*PfPDEβ*^688-1139^ | pET-*6HIS*-*SUMO*-*PfPDEβ*^688-1139^ driven by a *T7* promoter with a *KanR* drug selectable marker. | Expression and purification of recombinant PfPDEβ^688-1139^ from *E. coli*. | This work |
