# Supplementary material for: Functional Analysis of the Expanded Phosphodiesterase Gene Family in Toxoplasma gondii Tachyzoites
Source: mSphere. 2022 Feb 2;7(1):e00793-21. doi: 10.1128/msphere.00793-21 (PMC8809380; doi:10.1128/msphere.00793-21)
Supplement: TABLE S3 [file msphere.00793-21-st003.docx]

**Table S3** Oligonucleotides used in this study.

| **P#** | **ssDNA OligoNT Name** | **Sequence (5’ – 3’)** | **Usage** | **Source** |
| --- | --- | --- | --- | --- |
| P1 | sg202540(PDE1) 3'UTR | TAAATGGGGATGTCAAGTTGAGGAGAGAAGCGCCGCTCTGTTTTAGAGCTAGAAATAGC | HiFi assembly of p*SAG1:Cas9-GFP, U6:sg202540(PDE1) 3’UTR* | This work; IDT |
| P2 | sg293000(PDE2) 3'UTR | TAAATGGGGATGTCAAGTTgAGACGCCAAGGAACGGCGCAGTTTTAGAGCTAGAAATAGC | HiFi assembly of p*SAG1:Cas9-GFP, U6:sg293000(PDE2) 3’UTR* | This work; IDT |
| P3 | sg233065(PDE3) 3'UTR | TAAATGGGGATGTCAAGTTgACCTGCTGAAGTAGAAGAAGGTTTTAGAGCTAGAAATAGC | HiFi assembly of p*SAG1:Cas9-GFP, U6:sg233065(PDE3) 3’UTR* | This work; IDT |
| P4 | sg229405(PDE4) 3'UTR | TAAATGGGGATGTCAAGTTgTCACGAATCTTCCACACCATGTTTTAGAGCTAGAAATAGC | HiFi assembly of p*SAG1:Cas9-GFP, U6:sg229405(PDE4) 3’UTR* | This work; IDT |
| P5 | sg220420(PDE5) 3'UTR | TAAATGGGGATGTCAAGTTgCGCGAAGGCTTCAACGCCTGGTTTTAGAGCTAGAAATAGC | HiFi assembly of p*SAG1:Cas9-GFP, U6:sg220420(PDE5) 3’UTR* | This work; IDT |
| P6 | sg266920(PDE6) 3'UTR | TAAATGGGGATGTCAAGTTgTGAAGCCACCTGTTCTTCGTGTTTTAGAGCTAGAAATAGC | HiFi assembly of p*SAG1:Cas9-GFP, U6:sg266920(PDE6) 3’UTR* | This work; IDT |
| P7 | sg280410(PDE7) 3'UTR | TAAATGGGGATGTCAAGTTGTAAAGCAGTGCCCTGAGAAGTTTTAGAGCTAGAAATAGC | HiFi assembly of p*SAG1:Cas9-GFP, U6:sg280410(PDE7) 3’UTR* | This work; IDT |
| P8 | sg318675(PDE8) 3'UTR | TAAATGGGGATGTCAAGTTgCGAGCCAAAGGACAAAGTTGGTTTTAGAGCTAGAAATAGC | HiFi assembly of p*SAG1:Cas9-GFP, U6:sg318675(PDE8) 3’UTR* | This work; IDT |
| P9 | sg241880(PDE9) 3'UTR | TAAATGGGGATGTCAAGTTgAGCGAGGAGCTGGAACGTCGGTTTTAGAGCTAGAAATAGC | HiFi assembly of p*SAG1:Cas9-GFP, U6:sg241880(PDE9) 3’UTR* | This work; IDT |
| P10 | sg272650(PDE10) 3'UTR | TAAATGGGGATGTCAAGTTGAGATGAACCATGACACTCGGTTTTAGAGCTAGAAATAGC | HiFi assembly of p*SAG1:Cas9-GFP, U6:sg272650(PDE10) 3’UTR* | This work; IDT |
| P11 | sg224840(PDE11) 3'UTR | TAAATGGGGATGTCAAGTTGAGAATGTGCAAAACAATTCGTTTTAGAGCTAGAAATAGC | HiFi assembly of p*SAG1:Cas9-GFP, U6:sg224840(PDE11) 3’UTR* | This work; IDT |
| P12 | sg310520(PDE12) 3'UTR | TAAATGGGGATGTCAAGTTgCATCTAAAAACATCGCACACGTTTTAGAGCTAGAAATAGC | HiFi assembly of p*SAG1:Cas9-GFP, U6:sg310520(PDE12) 3’UTR* | This work; IDT |
| P13 | sg257080(PDE13) 3'UTR | TAAATGGGGATGTCAAGTTGAGAGCCAAAAGCGCTTTCAGTTTTAGAGCTAGAAATAGC | HiFi assembly of p*SAG1:Cas9-GFP, U6:sg257080(PDE13) 3’UTR* | This work; IDT |
| P14 | sg228500(PDE14) 3'UTR | TAAATGGGGATGTCAAGTTgAGACCACCGAGTCAGCTAGGGTTTTAGAGCTAGAAATAGC | HiFi assembly of p*SAG1:Cas9-GFP, U6:sg228500(PDE14) 3’UTR* | This work; IDT |
| P15 | sg233040(PDE15) 3'UTR | TAAATGGGGATGTCAAGTTgCCTGAAATGGTATTACGAAGGTTTTAGAGCTAGAAATAGC | HiFi assembly of p*SAG1:Cas9-GFP, U6:sg233040(PDE15) 3’UTR* | This work; IDT |
| P16 | sg258508(PDE16) 3'UTR | TAAATGGGGATGTCAAGTTgCTTTGGAACGACTACAGTATGTTTTAGAGCTAGAAATAGC | HiFi assembly of p*SAG1:Cas9-GFP, U6:sg258508(PDE16) 3’UTR* | This work; IDT |
| P17 | sg257945(PDE17) 3'UTR | TAAATGGGGATGTCAAGTTgTTCTGGTGCCCCTCCTCAGCGTTTTAGAGCTAGAAATAGC | HiFi assembly of p*SAG1:Cas9-GFP, U6:sg257945(PDE17) 3’UTR* | This work; IDT |
| P18 | sg226755(PDE18) 3'UTR | TAAATGGGGATGTCAAGTTgAGAACGAAGCAGATTGACAAGTTTTAGAGCTAGAAATAGC | HiFi assembly of p*SAG1:Cas9-GFP, U6:sg226755(PDE18) 3’UTR* | This work; IDT |
| P19 | pU6_Seq_F | CTCTCGAATGTCTTCTCTGACAAG | Sanger sequencing primer for p*SAG1:Cas9-GFP, U6:sg”GOI” 3’ UTR* | This work; IDT |
| P20 | tag202540(PDE1) F | CCAACAAAGTGGAAGAAGAGTATCTGAAGGCACATCCGCCGCTAGCAAGGGCTCGGGCTC | Amplify *TgPDE1-mAID-3HA, DHFR-TS:HXGPRT* tagging amplicon from p*TUB1:YFP-mAID-3HA, DHFR-TS:HXGPRT* | This work; IDT |
| P21 | tag202540(PDE1) R | TAGATAGATCGATAGATCGATGGATCGTTTGAGGTCTGCTATAGGGCGAATTGGAGCTCC |  | This work; IDT |
| P22 | tag293000(PDE2) F | CGAGGAGTTCGACGGCTATCGACCGCCCAGCTTGGACGTAGCTAGCAAGGGCTCGGGCTC | Amplify *TgPDE2-mAID-3HA, DHFR-TS:HXGPRT* tagging amplicon from p*TUB1:YFP-mAID-3HA, DHFR-TS:HXGPRT* | This work; IDT |
| P23 | tag293000(PDE2) R | CCATCTGCCATTCTTCTTTCTCGTTGACGCGCTCCGACCTATAGGGCGAATTGGAGCTCC |  | This work; IDT |
| P24 | tag233065(PDE3) F | AAACAAATGGAAATCCATCATCGATGGGCATGAACGTCGGGCTAGCAAGGGCTCGGGCTC | Amplify *TgPDE3-mAID-3HA, DHFR-TS:HXGPRT* tagging amplicon from p*TUB1:YFP-mAID-3HA, DHFR-TS:HXGPRT* | This work; IDT |
| P25 | tag233065(PDE3) R | GGTCGAAAGGAGTCTCTCTGTGTCAGAATCTTTGTTCAGAATAGGGCGAATTGGAGCTCC |  | This work; IDT |
| P26 | tag229405(PDE4) F | GGAGCTCCGGGCCAACGCGAATCCATTATCGGAGGTCGACGCTAGCAAGGGCTCGGGCTC | Amplify *TgPDE4-mAID-3HA, DHFR-TS:HXGPRT* tagging amplicon from p*TUB1:YFP-mAID-3HA, DHFR-TS:HXGPRT* | This work; IDT |
| P27 | tag229405(PDE4) R | GGTCGTCCTCCGGTCAAGACCAGTTTTCCGGTACATCCCGATAGGGCGAATTGGAGCTCC |  | This work; IDT |
| P28 | tag220420(PDE5) F | GAACTCTGCTGAGGCCCTGCAGGAAGGATTTAAAACGGTCGCTAGCAAGGGCTCGGGCTC | Amplify *TgPDE5-mAID-3HA, DHFR-TS:HXGPRT* tagging amplicon from p*TUB1:YFP-mAID-3HA, DHFR-TS:HXGPRT* | This work; IDT |
| P29 | tag220420(PDE5) R | AAAAATACAAAAGCATTCTCGGAGGGCGCAAGGGACACCAATAGGGCGAATTGGAGCTCC |  | This work; IDT |
| P30 | tag266920(PDE6) F | CAACACTCCGTGTGGTGGCGAGGCAAACACCTTGGAGTCGGCTAGCAAGGGCTCGGGCTC | Amplify *TgPDE6-mAID-3HA, DHFR-TS:HXGPRT* tagging amplicon from p*TUB1:YFP-mAID-3HA, DHFR-TS:HXGPRT* | This work; IDT |
| P31 | tag266920(PDE6) R | AACACCCGTAACACAGATGCACTCCCATAAAGCTGCTTTTATAGGGCGAATTGGAGCTCC |  | This work; IDT |
| P32 | tag280410(PDE7) F | GGCTTCTCTGGAACCGGCTGGTCTAGAAGTTGGAGGAGCGGCTAGCAAGGGCTCGGGCTC | Amplify *TgPDE7-mAID-3HA, DHFR-TS:HXGPRT* tagging amplicon from p*TUB1:YFP-mAID-3HA, DHFR-TS:HXGPRT* | This work; IDT |
| P33 | tag280410(PDE7) R | TAATCCGGCTGTCTGTCTCTTCTCTCTTGGGTCTGAACCGATAGGGCGAATTGGAGCTCC |  | This work; IDT |
| P34 | tag318675(PDE8) F | TTTTACATCGAATAGTCTTTTCGATGGGCTTAATCGGTCCGCTAGCAAGGGCTCGGGCTC | Amplify *TgPDE8-mAID-3HA, DHFR-TS:HXGPRT* tagging amplicon from p*TUB1:YFP-mAID-3HA, DHFR-TS:HXGPRT* | This work; IDT |
| P35 | tag318675(PDE8) R | ACCAGAAGAATTCACGGCCGTCGCTCGACAGCAGGCACCGATAGGGCGAATTGGAGCTCC |  | This work; IDT |
| P36 | tag241880(PDE9) F | CAGGAAGGACCTTCTCCATTTCGAATCGCGACTGACGCATGCTAGCAAGGGCTCGGGCTC | Amplify *TgPDE9-mAID-3HA, DHFR-TS:HXGPRT* tagging amplicon from p*TUB1:YFP-mAID-3HA, DHFR-TS:HXGPRT* | This work; IDT |
| P37 | tag241880(PDE9) R | TTCTCCCGACCGTTTTTCAACAAGATCAGCATGTCTTCCTATAGGGCGAATTGGAGCTCC |  | This work; IDT |
| P38 | tag272650(PDE10) F | GGAAGGGGCGCAAGACGAAGTCGACCAGGCAAGCGCAAAGGCTAGCAAGGGCTCGGGCTC | Amplify *TgPDE10-mAID-3HA, DHFR-TS:HXGPRT* tagging amplicon from p*TUB1:YFP-mAID-3HA, DHFR-TS:HXGPRT* | This work; IDT |
| P39 | tag272650(PDE10) R | GACTTCAACACGCAGACGTCCTCTCTACCTCTTCCTCCCTATAGGGCGAATTGGAGCTCC |  | This work; IDT |
| P40 | tag224840(PDE11) F | GACGATGAAGAATCCAGAACACCACGCGTCTCAGAAGCAAGCTAGCAAGGGCTCGGGCTC | Amplify *TgPDE11-mAID-3HA, DHFR-TS:HXGPRT* tagging amplicon from p*TUB1:YFP-mAID-3HA, DHFR-TS:HXGPRT* | This work; IDT |
| P41 | tag224840(PDE11) R | ACGGAACGAGAAATGTCAAAGTCAGGGCCAAGGAGGCCGCATAGGGCGAATTGGAGCTCC |  | This work; IDT |
| P42 | tag310520(PDE12) F | AAGTGCCTGCGAGTCCCTTAGAGTGGGGCCGCGGGAAGTGGCTAGCAAGGGCTCGGGCTC | Amplify *TgPDE12-mAID-3HA, DHFR-TS:HXGPRT* tagging amplicon from p*TUB1:YFP-mAID-3HA, DHFR-TS:HXGPRT* | This work; IDT |
| P43 | tag310520(PDE12) R | TTCGGCGGACTACGTCTGTGCAAATGTCCTGCACCTGCCAATAGGGCGAATTGGAGCTCC |  | This work; IDT |
| P44 | tag257080(PDE13) F | GAAGCGTCGCGACGAGAAGAAGCCCGAGAAGAGCAAGACAGCTAGCAAGGGCTCGGGCTC | Amplify *TgPDE13-mAID-3HA, DHFR-TS:HXGPRT* tagging amplicon from p*TUB1:YFP-mAID-3HA, DHFR-TS:HXGPRT* | This work; IDT |
| P45 | tag257080(PDE13) R | ATGCCGCATTCCCGCAAATGGCTGTTGGTAGATCTGTCCCATAGGGCGAATTGGAGCTCC |  | This work; IDT |
| P46 | tag228500(PDE14) F | GCAGAAAAACAGGTGTGTACCTTTGATTATCTTCCCCACCGCTAGCAAGGGCTCGGGCTC | Amplify *TgPDE14-mAID-3HA, DHFR-TS:HXGPRT* tagging amplicon from p*TUB1:YFP-mAID-3HA, DHFR-TS:HXGPRT* | This work; IDT |
| P47 | tag228500(PDE14) R | AGCTATGTGGGCGCCAAGAAACTCGAAGGTCAGGAGTATTATAGGGCGAATTGGAGCTCC |  | This work; IDT |
| P48 | tag233040(PDE15) F | TCAAGATCAAGAACAAGCGGACTATGCGTATCCAGAAGGTGCTAGCAAGGGCTCGGGCTC | Amplify *TgPDE15-mAID-3HA, DHFR-TS:HXGPRT* tagging amplicon from p*TUB1:YFP-mAID-3HA, DHFR-TS:HXGPRT* | This work; IDT |
| P49 | tag233040(PDE15) R | TGGAACTATCACACACAGTTAACTCAATACCCGTCATCCTATAGGGCGAATTGGAGCTCC |  | This work; IDT |
| P50 | tag258508(PDE16) F | AAATCAGAAGAACCTCATGCAAAAACTGGACAAAAGTGATGCTAGCAAGGGCTCGGGCTC | Amplify *TgPDE16-mAID-3HA, DHFR-TS:HXGPRT* tagging amplicon from p*TUB1:YFP-mAID-3HA, DHFR-TS:HXGPRT* | This work; IDT |
| P51 | tag258508(PDE16) R | AATTTGTAAACATTGAAGCGGTGGATATTCACACATCCCGATAGGGCGAATTGGAGCTCC |  | This work; IDT |
| P52 | tag257945(PDE17) F | CCGGAGGCACCTCAAACGATGGTCAACGATCTCACCCTGCGCTAGCAAGGGCTCGGGCTC | Amplify *TgPDE17-mAID-3HA, DHFR-TS:HXGPRT* tagging amplicon from p*TUB1:YFP-mAID-3HA, DHFR-TS:HXGPRT* | This work; IDT |
| P53 | tag257945(PDE17) R | ACGGCTGACGGTAAATTCTTGGGCGTGTGCGCTGCTCTTTATAGGGCGAATTGGAGCTCC |  | This work; IDT |
| P54 | tag226755(PDE18) F | GGAACTGTTGCACGTCGAAAAAAGGGTGTGCGAAACAGTGGCTAGCAAGGGCTCGGGCTC | Amplify *TgPDE18-mAID-3HA, DHFR-TS:HXGPRT* tagging amplicon from p*TUB1:YFP-mAID-3HA, DHFR-TS:HXGPRT* | This work; IDT |
| P55 | tag226755(PDE18) R | CCCTCAGTCCTCCTTGTTTCTTATCCTTCACAATCGCCCTATAGGGCGAATTGGAGCTCC |  | This work; IDT |
| P56 | 202540(PDE1)_3’ F | CCGCAGAAATCGCAAACAG | Forward primer for *TgPDE1* Diagnostic PCRs 1, 2 | This work; IDT |
| P57 | 202540(PDE1)_3’ R | TGTGTATGTAACAACGGACATAGA | Reverse primer for *TgPDE1* Diagnostic PCR 1 | This work; IDT |
| P58 | 293000(PDE2)_3’ F | GGGTTCCTACGTCTTTCTTTCA | Forward primer for *TgPDE2* Diagnostic PCRs 1, 2 | This work; IDT |
| P59 | 293000(PDE2)_3’ R | CTGAATGCGGAACGCAAATC | Reverse primer for *TgPDE2* Diagnostic PCR 1 | This work; IDT |
| P60 | 233065(PDE3)_3’ F | TGCTGCACATTTGAGCGT | Forward primer for *TgPDE3* Diagnostic PCRs 1, 2 | This work; IDT |
| P61 | 233065(PDE3)_3’ R | CGACCCGCTAAGAAATTCATGT | Reverse primer for *TgPDE3* Diagnostic PCR 1 | This work; IDT |
| P62 | 229405(PDE4)_3’ F | CGACTGAGGCAACAGGAAA | Forward primer for *TgPDE4* Diagnostic PCRs 1, 2 | This work; IDT |
| P63 | 229405(PDE4)_3’ R | GAGATGTTCCGCCCATGAA | Reverse primer for *TgPDE4* Diagnostic PCR 1 | This work; IDT |
| P64 | 220420(PDE5)_3’ F | CCTCCGTTCTTCATTCCTTGT | Forward primer for *TgPDE5* Diagnostic PCRs 1, 2 | This work; IDT |
| P65 | 220420(PDE5)_3’ R | GTAACTCAGGAGAAAGCGTACC | Reverse primer for *TgPDE5* Diagnostic PCR 1 | This work; IDT |
| P66 | 266920(PDE6)_3’ F | CTGCCCGTCTCTACTACAGAAT | Forward primer for *TgPDE6* Diagnostic PCRs 1, 2 | This work; IDT |
| P67 | 266920(PDE6)_3’ R | CGCTGCGTACCAAACCTTTA | Reverse primer for *TgPDE6* Diagnostic PCR 1 | This work; IDT |
| P68 | 280410(PDE7)_3’ F | TAAACCCTCAGCTGCCAATC | Forward primer for *TgPDE7* Diagnostic PCRs 1, 2 | This work; IDT |
| P69 | 280410(PDE7)_3’ R | TATCTCAGCTTCTCGCCTCT | Reverse primer for *TgPDE7* Diagnostic PCR 1 | This work; IDT |
| P70 | 318675(PDE8)_3’ F | GAGGGACACGACAATGAGAAG | Forward primer for *TgPDE8* Diagnostic PCRs 1, 2 | This work; IDT |
| P71 | 318675(PDE8)_3’ R | TGCCTGTTTCCTGACATACTG | Reverse primer for *TgPDE8* Diagnostic PCR 1 | This work; IDT |
| P72 | 241880(PDE9)_3’ F | CACGAGAGGAACAGACAAGAC | Forward primer for *TgPDE9* Diagnostic PCRs 1, 2 | This work; IDT |
| P73 | 241880(PDE9)_3’ R | GATACCGAGACATCTTCCTCAAC | Reverse primer for *TgPDE9* Diagnostic PCR 1 | This work; IDT |
| P74 | 272650(PDE10)_3’ F | CTCTCGCGCAACACCTC | Forward primer for *TgPDE10* Diagnostic PCRs 1, 2 | This work; IDT |
| P75 | 272650(PDE10)_3’ R | CTGCAGGCCAGTCTTCTG | Reverse primer for *TgPDE10* Diagnostic PCR 1 | This work; IDT |
| P76 | 224840(PDE11)_3’ F | CTGAATTGGTGCATGGTTTCTT | Forward primer for *TgPDE11* Diagnostic PCRs 1, 2 | This work; IDT |
| P77 | 224840(PDE11)_3’ R | TCTGTCACGAACCTAGGACT | Reverse primer for *TgPDE11* Diagnostic PCR 1 | This work; IDT |
| P78 | 310520(PDE12)_3’ F | TTCTCGATTACGGAGGCTAC | Forward primer for *TgPDE12* Diagnostic PCRs 1, 2 | This work; IDT |
| P79 | 310520(PDE12)_3’ R | AAACGTTCCTAGCATTGTGAG | Reverse primer for *TgPDE12* Diagnostic PCR 1 | This work; IDT |
| P80 | 257080(PDE13)_3’ F | CGCAGACGAAGCAGGATT | Forward primer for *TgPDE13* Diagnostic PCRs 1, 2 | This work; IDT |
| P81 | 257080(PDE13)_3’ R | ACGACAACTTGAGTGACAGTG | Reverse primer for *TgPDE13* Diagnostic PCR 1 | This work; IDT |
| P82 | 228500(PDE14)_3’ F | TTGCCTTTATGTGTTGCAGATG | Forward primer for *TgPDE14* Diagnostic PCRs 1, 2 | This work; IDT |
| P83 | 228500(PDE14)_3’ R | CTACGGCCTTGCGATCATTA | Reverse primer for *TgPDE14* Diagnostic PCR 1 | This work; IDT |
| P84 | 233040(PDE15)_3’ F | GGAGCTTTCCAGGCGTATT | Forward primer for *TgPDE15* Diagnostic PCRs 1, 2 | This work; IDT |
| P85 | 233040(PDE15)_3’ R | AAATGCATACCGCCCTTACA | Reverse primer for *TgPDE15* Diagnostic PCR 1 | This work; IDT |
| P86 | 258508(PDE16)_3’ F | AGCCACTGATGACGGTTT | Forward primer for *TgPDE16* Diagnostic PCRs 1, 2 | This work; IDT |
| P87 | 258508(PDE16)_3’ R | GATACAACACAACACTTGATCCC | Reverse primer for *TgPDE16* Diagnostic PCR 1 | This work; IDT |
| P88 | 257945(PDE17)_3’ F | CCATGGCCGAGGAACAG | Forward primer for *TgPDE17* Diagnostic PCRs 1, 2 | This work; IDT |
| P89 | 257945(PDE17)_3’ R | CATAAGAGTGGAACGGAGAAGG | Reverse primer for *TgPDE17* Diagnostic PCR 1 | This work; IDT |
| P90 | 226755(PDE18)_3’ F | GCGAGAAGCCAGTAAAGAGAA | Forward primer for *TgPDE18* Diagnostic PCRs 1, 2 | This work; IDT |
| P91 | 226755(PDE18)_3’ R | GTGCAGCTTTGACGCATAAC | Reverse primer for *TgPDE18* Diagnostic PCR 1 | This work; IDT |
| P92 | HXG 3' UTR R | GCTATTATACCCGTGTGTTACG | Reverse primer for all *TgPDE* Diagnostic PCR 2 | This work; IDT |
| P93 | pET-SUMO F | TAGAGACAAGCTTAGGTATTTATTCGGCG | Amplify vector backbone from pET-*6HIS-SUMO-hGSDMD* plasmid | This work; IDT |
| P94 | pET-SUMO R | ACCACCAATCTGTTCTCTGTGAG |  | This work; IDT |
| P95 | SUMO Seq F | CTGAAGATTTGGACATGGAGG | Sanger sequencing primers for confirmation of *TgPDE* plasmid sequences | This work; IDT |
| P96 | SUMO Seq R | GCATCACCCGACGCACTTTG |  | This work; IDT |
| P97 | 202540(PDE1) CAT F | ACAGAGAACAGATTGGTGGTTGCCACCAAACGCTGAGTC | Amplify sequence corresponding to TgPDE1^974-1670^ from RH cDNA with homology flanks to pET-*6HIS-SUMO* plasmid for HiFi assembly | This work; IDT |
| P98 | 202540(PDE1) CAT R | AATACCTAAGCTTGTCTCTAGGCGGATGTGCCTTCAGAT |  | This work; IDT |
| P99 | 293000(PDE2) CAT F | ACAGAGAACAGATTGGTGGTTTGCTAGCAACGCGTCTGTC | Amplify sequence corresponding to TgPDE2^1591-2238^ from RH cDNA with homology flanks to pET-*6HIS-SUMO* plasmid for HiFi assembly | This work; IDT |
| P100 | 293000(PDE2) CAT R | AATACCTAAGCTTGTCTCTATACGTCCAAGCTGGGCGGTCGATAGCC |  | This work; IDT |
| P101 | 233065(PDE3) CAT F | ACAGAGAACAGATTGGTGGTGGCTTTGCCGTTGCTCTCTAT | Amplify sequence corresponding to TgPDE3^559-820^ from RH cDNA with homology flanks to pET-*6HIS-SUMO* plasmid for HiFi assembly | This work; IDT |
| P102 | 233065(PDE3) CAT R | AATACCTAAGCTTGTCTCTATTTCCATTTGTTTCTGTTCTTTCG |  | This work; IDT |
| P103 | 229405(PDE4) CAT F | ACAGAGAACAGATTGGTGGTCTCTACGAGATGGGATCGGAA | Amplify sequence corresponding to TgPDE4^580-1033^ from RH cDNA with homology flanks to pET-*6HIS-SUMO* plasmid for HiFi assembly | This work; IDT |
| P104 | 229405(PDE4) CAT R | AATACCTAAGCTTGTCTCTATAATGGATTCGCGTTGGCCC |  | This work; IDT |
| P105 | 220420(PDE5) CAT F | ACAGAGAACAGATTGGTGGTTGGCGTAACTTCCTCAGAGAAG | Amplify sequence corresponding to TgPDE5^721-1023^ from RH cDNA with homology flanks to pET-*6HIS-SUMO* plasmid for HiFi assembly | This work; IDT |
| P106 | 220420(PDE5) CAT R | AATACCTAAGCTTGTCTCTATTTCGTCTTTATTCGGTTCCAGG |  | This work; IDT |
| P107 | 266920(PDE6) CAT F | ACAGAGAACAGATTGGTGGTGGGATCCTCGTGAATGAGCT | Amplify sequence corresponding to TgPDE6^447-1065^ from RH cDNA with homology flanks to pET-*6HIS-SUMO* plasmid for HiFi assembly | This work; IDT |
| P108 | 266920(PDE6) CAT R | AATACCTAAGCTTGTCTCTACGACTCCAAGGTGTTTGCC |  | This work; IDT |
| P109 | 280410(PDE7) CAT F | ACAGAGAACAGATTGGTGGTATTTTCGCGTTGATGCAGGA | Amplify sequence corresponding to TgPDE7^675-1085^ from RH cDNA with homology flanks to pET-*6HIS-SUMO* plasmid for HiFi assembly | This work; IDT |
| P110 | 280410(PDE7) CAT R | AATACCTAAGCTTGTCTCTACGCTCCTCCAACTTCTAGACC |  | This work; IDT |
| P111 | 318675(PDE8) CAT F | ACAGAGAACAGATTGGTGGTGCTCTCGAGCTTGTAGAGGAAG | Amplify sequence corresponding to TgPDE8^522-1123^ from RH cDNA with homology flanks to pET-*6HIS-SUMO* plasmid for HiFi assembly | This work; IDT |
| P112 | 318675(PDE8) CAT R | AATACCTAAGCTTGTCTCTAGGACCGATTAAGCCCATCGA |  | This work; IDT |
| P113 | 241880(PDE9) CAT F | ACAGAGAACAGATTGGTGGTTCTGAGGGCATGCTTATCAGG | Amplify sequence corresponding to TgPDE9^650-1281^ from RH cDNA with homology flanks to pET-*6HIS-SUMO* plasmid for HiFi assembly | This work; IDT |
| P114 | 241880(PDE9) CAT R | AATACCTAAGCTTGTCTCTAATGCGTCAGTCGCGATTC |  | This work; IDT |
| P115 | 272650(PDE10) CAT F | ACAGAGAACAGATTGGTGGTCTGGATGCCCTCCGAGTC | Amplify sequence corresponding to TgPDE10^814-1294^ from RH cDNA with homology flanks to pET-*6HIS-SUMO* plasmid for HiFi assembly | This work; IDT |
| P116 | 272650(PDE10) CAT R | AATACCTAAGCTTGTCTCTACTTTGCGCTTGCCTGGTC |  | This work; IDT |
| P117 | 224840(PDE11) CAT F | ACAGAGAACAGATTGGTGGTGAGAAGCTTCTCGAAAGAATCCTTG | Amplify sequence corresponding to TgPDE11^540-1324^ from RH cDNA with homology flanks to pET-*6HIS-SUMO* plasmid for HiFi assembly | This work; IDT |
| P118 | 224840(PDE11) CAT R | AATACCTAAGCTTGTCTCTATTGCTTCTGAGACGCGTG |  | This work; IDT |
| P119 | 310520(PDE12) CAT F | ACAGAGAACAGATTGGTGGTAGTAGGGGGAAGCGAGAGGAAGAGC | Amplify sequence corresponding to TgPDE12^1112-1579^ from RH cDNA with homology flanks to pET-*6HIS-SUMO* plasmid for HiFi assembly | This work; IDT |
| P120 | 310520(PDE12) CAT R | AATACCTAAGCTTGTCTCTACACTTCCCGCGGCCCCAC |  | This work; IDT |
| P121 | 257080(PDE13) CAT F | ACAGAGAACAGATTGGTGGTGTGGCTGACATTCAAGGACAA | Amplify sequence corresponding to TgPDE13^1057-1656^ from RH cDNA with homology flanks to pET-*6HIS-SUMO-hGSDMD* plasmid for HiFi assembly | This work; IDT |
| P122 | 257080(PDE13) CAT R | AATACCTAAGCTTGTCTCTATGTCTTGCTCTTCTCGGGC |  | This work; IDT |
| P123 | 228500(PDE14) CAT F | ACAGAGAACAGATTGGTGGTGACTGGGATTTTCACGTCCT | Amplify sequence corresponding to TgPDE14^6-291^ from RH cDNA with homology flanks to pET-*6HIS-SUMO* plasmid for HiFi assembly | This work; IDT |
| P124 | 228500(PDE14) CAT R | AATACCTAAGCTTGTCTCTACAGCTGGGCAGGAAACATGA |  | This work; IDT |
| P125 | 233040(PDE15) CAT F | ACAGAGAACAGATTGGTGGTGAGCCTGCAGGCCAGGAA | Amplify sequence corresponding to TgPDE15^952-1731^ from RH cDNA with homology flanks to pET-*6HIS-SUMO* plasmid for HiFi assembly | This work; IDT |
| P126 | 233040(PDE15) CAT R | AATACCTAAGCTTGTCTCTAACCTTCTGGATACGCATAGTCCGC |  | This work; IDT |
| P127 | 258508(PDE16) CAT F | ACAGAGAACAGATTGGTGGTACTGCGACCGTTGGAGGTATG | Amplify sequence corresponding to TgPDE16^233-609^ from RH cDNA with homology flanks to pET-*6HIS-SUMO* plasmid for HiFi assembly | This work; IDT |
| P128 | 258508(PDE16) CAT R | AATACCTAAGCTTGTCTCTAATCACTTTTGTCCAGTTTTTGCATG |  | This work; IDT |
| P129 | 257945(PDE17) CAT F | ACAGAGAACAGATTGGTGGTCACCGTCGGCCCTCTCTAAGTG | Amplify sequence corresponding to TgPDE17^1405-1865^ from RH cDNA with homology flanks to pET-*6HIS-SUMO* plasmid for HiFi assembly | This work; IDT |
| P130 | 257945(PDE17) CAT R | AATACCTAAGCTTGTCTCTAGGATGCTTCGAAGTTTCTGCC |  | This work; IDT |
| P131 | 226755(PDE18) CAT F | ACAGAGAACAGATTGGTGGTGAGCACGTGCACTCGAGCC | Amplify sequence corresponding to TgPDE18^2700-3066^ from RH cDNA with homology flanks to pET-*6HIS-SUMO* plasmid for HiFi assembly | This work; IDT |
| P132 | 226755(PDE18) CAT R | AATACCTAAGCTTGTCTCTAATCGGAAGAACTAAACGCTTTACTC |  | This work; IDT |
| P133 | 1209500(PfPDEα) CAT F | ACAGAGAACAGATTGGTGGTATCAAAGAAAAAATCAAAAATTGTGATAAT | Amplify sequence corresponding to PfPDEα^540-954^ from *P. falciparum* cDNA with homology flanks to pET-*6HIS-SUMO* plasmid for HiFi assembly | This work; IDT |
| P134 | 1209500(PfPDEα) CAT R | AATACCTAAGCTTGTCTCTATTCAAATTTGATGAGCTCAAGT |  | This work; IDT |
| P135 | 1321500(PfPDEβ) CAT F | ACAGAGAACAGATTGGTGGTTGTTCGAATATTTTGGAACAATG | Amplify sequence corresponding to PfPDEβ^688-1139^ from *P. falciparum* cDNA with homology flanks to pET-*6HIS-SUMO* plasmid for HiFi assembly | This work; IDT |
| P136 | 1321500(PfPDEβ) CAT R | AATACCTAAGCTTGTCTCTAATCGGAAACATTTTTTATAAAAAATAGAGT |  | This work; IDT |

dsDNA OligoNT sequences continued on next page.

| **G#** | **dsDNA OligoNT (gBlock) Name** | **Sequence (5’ - 3’)** | **Usage** | **Source** |
| --- | --- | --- | --- | --- |
| G1 | 233065(PDE3) CAT gBlock | ACAGAGAACAGATTGGTGGTGGCTTTGCCGTTGCTCTCTATAAAAACTACGAACAGAATCCGTACCACAATTTCTTTCATGCCCTCAATGTCGCCCAAGTCTGCTGCCTGCTCATGGCCCTGCCAGACGTCGCGGCTCGGTTCCAACCTCTGGACTACTTTGTGCTCTCCGTCGCTGCTTTGGGCCATGACCTAGGCCACCCAGGCGCGAACAATCTCTTCGTCAATCGAAACGACTGCTTGCCCTCTCGTCTCTACCAGAACCGGTCTGTTCTCGAAAACTACCACGCGGCTCTACTTTTCCAAATTCTCCGGCACCCCCGGTTTAACGTATTCTGCTCCATTCCGCCTCAAGCCTTCTCGGCTTGCCGACAGCGCATCATCAGCGCGATTCTGTGGACCGACATGGCCAAGCATTTCGACATGGTCGCGCAACTCAAGGCGAAAATCGAGGACGAAATGGTGTTGACCGAAGGCATCATTGTCACGCTGCAAAAGCCGTATCTGGAGGGGCTGCTCCTCCACGCCTCCGACATTTCAAATCCCTTGCTCAGTTTCGATCTCTCCTTCGACTGGGCTGTCAGAGCCTGCGACGAATTCTTTCAGCAGAACAAACTCGAAGAGAAGCTCGGCCAGCCGCCGCACATGCCTACCTTCGCGGCATTCGACCTCTACAACGTCGCAAAATGCCAAGTGAATTTCATAGATTTTATTTGTCGCCCCCTCTTCGGTAGCCTCGCGCAGATGTTCCCGGCCCAGCTTGGAGACCGAGCTGCGGAGCTCCGAAAGAACAGAAACAAATGGAAATAGAGACAAGCTTAGGTATT | Clone TgGT1_233065 catalytic domain (TgPDE3^559-820^) into pET-*6HIS-SUMO* by HiFi assembly | This work; IDT |
| G2 | 233040(PDE15) CAT gBlock | ACAGAGAACAGATTGGTGGTGAGCCTGCAGGCCAGGAAGACGAACTCCCCACTGCTGAACGGACGACGGAGGAATTCAGATGGGGCAACATTCCCGAGTACACAGTGCCGAGCTGCATCACGGCGAGTGTTGCCAGGATGGATACAGTCTTGTGCTTGGCTCCGAGCATCGCGTCGGAGGCACGACTAAACACGTACAGAGAAAATGCTCCATCAATCGCTGTCGAGTTCATCTTCAGCTGGGACTTTGATATCTCCAGTCTCCAACCTGAAATGTCTGCGAAACTGGCGTACGAGTTGCTGATTTGGTGTGCAGATAAAGCGCACATTAGCGTTCCTCGGGAGACGTGCGAGAAGTTCGTCCTCTCTGTGCAGCAGAACTATTCTGCAATGCCTTTCCATAACTTCAACCACGCGTTGCATGCAGCTCAAGCAGTGATATTGATTGCAAAAGACCTGTCTTTTCAACATTGGTTTTCTTACGAAGATAAATTTATTATTATGCTCGCGGCGCTGGGTCATGACATGGGCCACCCTGGAGTATCAAATGAGTTTCTGATTAGCATGCGATCGTTCACGAGCATCTTGTTCAACGAAACGGCCGTTCTGGAGAATTACCACACCTTGCTTTACATGGATCTTTTGAAAAACTCTGATCTGGACATTTTGAAAATGCTCCAGCCCGAGGCCGTCCAGCGGGCGCGACAGAGAATCATCGCAGCCATCTTGGCCACCGATCGCGCGTTAGACATGAAACTCATTGACCTTCTGAACGATGTCAAAAGCAAAAACGAGAAAGTGCCGGTACCCGCCGCGCTCCTCGATCCAAATGTCAGGGACGCCTGTCTCATTCATGCGGCTGACCACGCTCTCTCGCTGCTCAACTTTCCACTCCATCGGAAGTGGGCTGAGAAAATGGCGATCGAAATGCACTTCCAGAACACTATGGACGAAGCCTTAAAGCTGCCCAAGAGCTACTGCAGCCTCAGTAGATTAACAGAAGCATCCCTTGCTGGATCCCAAGTGACGATGATAGATACACACTACCTGCCATTCTTCAGCGCCCTCGCTTGGTATTTCCCTGGCGACCTCGATATGAGAGTTGCTGTGATGAAAGCCAACGCTGCGTTCTGGCAGCATCTTCGTGATGAAGAAATGAGGAAAACAGTCGCACTGAATGACAATCCGGAGCAAGAAGATTGGAGGAGTGCAAAACTGCAAGTAATCTCTAGCAAATTCGTCGAGAATAGAGAGAAAATGCACCAGAAAGCGGCGGAAATGATGCAGGCCGAGGCTACAGATGTTTTCTTTGACCCTTACCCAATGATAGTAGATCAAAGTGGCAACGAGGGCGAAGAGGACTCCTTCGCGAAAGGGGTGATGCGCGAGAAATCCGGAATGGGGGGGTACGCAATAGAGAGCAGACGTCGGTCAGCTGATTCATCCAGGGGAGCGGAACGGCGGGAATCAGATGCTGAAGCTCATCATTCGGAAGCAACGCATGCCCCGAAACCAGACGAAAAGTCTCAGTTAGACAAGAATGGGCAGCGACAGTCCAACAGTTCGGGTCTGGAACAAGGCAGTAATGCCGAGGTATCCTGCTTCCAGCGCGCAAGCCTGCGGGCTCGTTTCGTCGATGCTGATGTGACCATTCCGGCAACAAAGCAGCCAGTTCTGAGTGAAACACATGCGTCTGCAACCAATGTCACTTCTAGAGGGGAGCAAGGAAATGAAAAGGGAAACCTTGACAGCCCAGAGCAAACCACGAAAATAGGAGACGAAGAGAAGGAAGCGAGAACAGACGAGATGGTCGGAGATCCGGAAGAAACACGTGAGAAACATGATCCTCCAACAGTATCACACTTCGAAGACAGAAGTGACCATAGACAAGTCGATGCCCCAGATTCACTCCCAGAAGCAGCACCTAACGTAGGGAGCCAAAACCCCAATTCAGCAGGAACGCATCCAGACACAAGAGCCACTTCTTCAGTGTCTGTCCCGAGTAACGTCGCTGATGAAGAGGTTGCAGAACGGGTGCCGGCACCTGTTCCGGCGGACCAGTCGTTATCTAGAACTTTACTCCAAAGTGACGAAGCATCTCAACGAAAGAACCAAACGAAGAGTCTTTCAAAAGCTGAAAGCGTGGACACAGAGGAAATGTCGGCTTTCGTGCTGGACGAAGTCTCTGGGTTCTTTTACGACCAGCAAGGCTACGTGTACGACCAACAAGGAAACCTCAGAGGTTATTTGGATGCCAGTGGAGCTTTCCAGGCGTATTCGGAAAGTGAACTGCAGTATTACGCGGAAACCATGGCAAGAGTTCAAGATCAAGAACAAGCGGACTATGCGTATCCAGAAGGTTAGAGACAAGCTTAGGTATT | Clone TgGT1_233040 catalytic domain (TgPDE15^952-1731^) into pET-*6HIS-SUMO* by HiFi assembly | This work; IDT |

| **G#** | **dsDNA OligoNT (gBlock) Name** | **Sequence (5’ - 3’)** | **Usage** | **Source** |
| --- | --- | --- | --- | --- |
| G1 | 233065(PDE3) CAT gBlock | ACAGAGAACAGATTGGTGGTGGCTTTGCCGTTGCTCTCTATAAAAACTACGAACAGAATCCGTACCACAATTTCTTTCATGCCCTCAATGTCGCCCAAGTCTGCTGCCTGCTCATGGCCCTGCCAGACGTCGCGGCTCGGTTCCAACCTCTGGACTACTTTGTGCTCTCCGTCGCTGCTTTGGGCCATGACCTAGGCCACCCAGGCGCGAACAATCTCTTCGTCAATCGAAACGACTGCTTGCCCTCTCGTCTCTACCAGAACCGGTCTGTTCTCGAAAACTACCACGCGGCTCTACTTTTCCAAATTCTCCGGCACCCCCGGTTTAACGTATTCTGCTCCATTCCGCCTCAAGCCTTCTCGGCTTGCCGACAGCGCATCATCAGCGCGATTCTGTGGACCGACATGGCCAAGCATTTCGACATGGTCGCGCAACTCAAGGCGAAAATCGAGGACGAAATGGTGTTGACCGAAGGCATCATTGTCACGCTGCAAAAGCCGTATCTGGAGGGGCTGCTCCTCCACGCCTCCGACATTTCAAATCCCTTGCTCAGTTTCGATCTCTCCTTCGACTGGGCTGTCAGAGCCTGCGACGAATTCTTTCAGCAGAACAAACTCGAAGAGAAGCTCGGCCAGCCGCCGCACATGCCTACCTTCGCGGCATTCGACCTCTACAACGTCGCAAAATGCCAAGTGAATTTCATAGATTTTATTTGTCGCCCCCTCTTCGGTAGCCTCGCGCAGATGTTCCCGGCCCAGCTTGGAGACCGAGCTGCGGAGCTCCGAAAGAACAGAAACAAATGGAAATAGAGACAAGCTTAGGTATT | Clone TgGT1_233065 catalytic domain (TgPDE3^559-820^) into pET-*6HIS-SUMO* by HiFi assembly | This work; IDT |
| G2 | 233040(PDE15) CAT gBlock | ACAGAGAACAGATTGGTGGTGAGCCTGCAGGCCAGGAAGACGAACTCCCCACTGCTGAACGGACGACGGAGGAATTCAGATGGGGCAACATTCCCGAGTACACAGTGCCGAGCTGCATCACGGCGAGTGTTGCCAGGATGGATACAGTCTTGTGCTTGGCTCCGAGCATCGCGTCGGAGGCACGACTAAACACGTACAGAGAAAATGCTCCATCAATCGCTGTCGAGTTCATCTTCAGCTGGGACTTTGATATCTCCAGTCTCCAACCTGAAATGTCTGCGAAACTGGCGTACGAGTTGCTGATTTGGTGTGCAGATAAAGCGCACATTAGCGTTCCTCGGGAGACGTGCGAGAAGTTCGTCCTCTCTGTGCAGCAGAACTATTCTGCAATGCCTTTCCATAACTTCAACCACGCGTTGCATGCAGCTCAAGCAGTGATATTGATTGCAAAAGACCTGTCTTTTCAACATTGGTTTTCTTACGAAGATAAATTTATTATTATGCTCGCGGCGCTGGGTCATGACATGGGCCACCCTGGAGTATCAAATGAGTTTCTGATTAGCATGCGATCGTTCACGAGCATCTTGTTCAACGAAACGGCCGTTCTGGAGAATTACCACACCTTGCTTTACATGGATCTTTTGAAAAACTCTGATCTGGACATTTTGAAAATGCTCCAGCCCGAGGCCGTCCAGCGGGCGCGACAGAGAATCATCGCAGCCATCTTGGCCACCGATCGCGCGTTAGACATGAAACTCATTGACCTTCTGAACGATGTCAAAAGCAAAAACGAGAAAGTGCCGGTACCCGCCGCGCTCCTCGATCCAAATGTCAGGGACGCCTGTCTCATTCATGCGGCTGACCACGCTCTCTCGCTGCTCAACTTTCCACTCCATCGGAAGTGGGCTGAGAAAATGGCGATCGAAATGCACTTCCAGAACACTATGGACGAAGCCTTAAAGCTGCCCAAGAGCTACTGCAGCCTCAGTAGATTAACAGAAGCATCCCTTGCTGGATCCCAAGTGACGATGATAGATACACACTACCTGCCATTCTTCAGCGCCCTCGCTTGGTATTTCCCTGGCGACCTCGATATGAGAGTTGCTGTGATGAAAGCCAACGCTGCGTTCTGGCAGCATCTTCGTGATGAAGAAATGAGGAAAACAGTCGCACTGAATGACAATCCGGAGCAAGAAGATTGGAGGAGTGCAAAACTGCAAGTAATCTCTAGCAAATTCGTCGAGAATAGAGAGAAAATGCACCAGAAAGCGGCGGAAATGATGCAGGCCGAGGCTACAGATGTTTTCTTTGACCCTTACCCAATGATAGTAGATCAAAGTGGCAACGAGGGCGAAGAGGACTCCTTCGCGAAAGGGGTGATGCGCGAGAAATCCGGAATGGGGGGGTACGCAATAGAGAGCAGACGTCGGTCAGCTGATTCATCCAGGGGAGCGGAACGGCGGGAATCAGATGCTGAAGCTCATCATTCGGAAGCAACGCATGCCCCGAAACCAGACGAAAAGTCTCAGTTAGACAAGAATGGGCAGCGACAGTCCAACAGTTCGGGTCTGGAACAAGGCAGTAATGCCGAGGTATCCTGCTTCCAGCGCGCAAGCCTGCGGGCTCGTTTCGTCGATGCTGATGTGACCATTCCGGCAACAAAGCAGCCAGTTCTGAGTGAAACACATGCGTCTGCAACCAATGTCACTTCTAGAGGGGAGCAAGGAAATGAAAAGGGAAACCTTGACAGCCCAGAGCAAACCACGAAAATAGGAGACGAAGAGAAGGAAGCGAGAACAGACGAGATGGTCGGAGATCCGGAAGAAACACGTGAGAAACATGATCCTCCAACAGTATCACACTTCGAAGACAGAAGTGACCATAGACAAGTCGATGCCCCAGATTCACTCCCAGAAGCAGCACCTAACGTAGGGAGCCAAAACCCCAATTCAGCAGGAACGCATCCAGACACAAGAGCCACTTCTTCAGTGTCTGTCCCGAGTAACGTCGCTGATGAAGAGGTTGCAGAACGGGTGCCGGCACCTGTTCCGGCGGACCAGTCGTTATCTAGAACTTTACTCCAAAGTGACGAAGCATCTCAACGAAAGAACCAAACGAAGAGTCTTTCAAAAGCTGAAAGCGTGGACACAGAGGAAATGTCGGCTTTCGTGCTGGACGAAGTCTCTGGGTTCTTTTACGACCAGCAAGGCTACGTGTACGACCAACAAGGAAACCTCAGAGGTTATTTGGATGCCAGTGGAGCTTTCCAGGCGTATTCGGAAAGTGAACTGCAGTATTACGCGGAAACCATGGCAAGAGTTCAAGATCAAGAACAAGCGGACTATGCGTATCCAGAAGGTTAGAGACAAGCTTAGGTATT | Clone TgGT1_233040 catalytic domain (TgPDE15^952-1731^) into pET-*6HIS-SUMO* by HiFi assembly | This work; IDT |
